# Birth prevalence of congenital heart disease in China, 1980–2019: a systematic review and meta-analysis of 617 studies

**DOI:** 10.1007/s10654-020-00653-0

**Published:** 2020-06-09

**Authors:** Lijuan Zhao, Lizhang Chen, Tubao Yang, Tingting Wang, Senmao Zhang, Letao Chen, Ziwei Ye, Liu Luo, Jiabi Qin

**Affiliations:** 1 Department of Epidemiology and Health Statistics, Xiangya School of Public Health, Central South University, 110 Xiangya Road, Changsha, 410078 Hunan China; 2 Hunan Provincial Key Laboratory of Clinical Epidemiology, Changsha, Hunan China; 3 National Health Commission Key Laboratory for Birth Defect Research and Prevention, Changsha, Hunan China; 4 Guangdong Cardiovascular Institute, Guangdong Provincial People’s Hospital, Academy of Medical Sciences, Guangzhou, Guangdong China

**Keywords:** Congenital heart diseases, Prevalence, Chinese, Spatial analysis, Meta-analysis, Systematic review

## Abstract

To assess the birth prevalence and spatial distribution of congenital heart disease (CHD) in China by conducting a complete overview and using spatial epidemiological methods. Unrestricted searches were conducted on seven electronic databases, with an end-date parameter of May 2019. Data on the birth prevalence of CHD and its subtypes were collected and combined using either the random-effect model or fixed-effect model. Subgroup sensitivity analyses were performed to explore potential heterogeneity moderators. The three-dimensional trend analysis and a visualization of CHD birth prevalence among different provinces were performed to describe the spatial distribution characteristics. Total 617 studies involving 76,961,354 births and 201,934 CHD individuals were included. Overall, total CHD birth prevalence increased continuously over time, from 0.201‰ in 1980–1984 to 4.905‰ in 2015–2019. The study on the high-income provinces, population-based monitoring model, male births, and urban regions reported a significantly higher prevalence of total CHD compared with upper-middle-income provinces, hospital-based monitoring model, female births, and rural regions, respectively. National CHD birth prevalence increased gradually from western region to eastern region, but decreased gradually from southern to northern region. Relevant heterogeneity moderators including gender, geographic region, income levels, and monitoring models have been identified by subgroup analyses. Sensitivity analysis yielded consistent results. Total CHD birth prevalence in China increases continuously in the past 40 years. Significant differences in gender, geographical regions, income levels, and monitoring models were found. In the future, population wide prospective birth defect registries covering the entire Chinese population need to determine the exact birth prevalence.

**Electronic supplementary material:**

The online version of this article (10.1007/s10654-020-00653-0) contains supplementary material, which is available to authorized users.

## Introduction

Congenital heart disease (CHD) is typically defined as clinically significant structural heart and/or great vessels disease present at birth [1]. CHD is the most common congenital anomalies worldwide, accounting for nearly one-third of all major congenital anomalies, and resulting in a huge health, social, and economic burden [2]. Although the prognosis and life quality of children with CHD continues to improve with innovative medical techniques, the effects of CHD on children are still lifelong. In order to effectively reduce the burden of CHD and to guide policy and advocacy efforts, accurate data on the CHD birth prevalence are needed.

According to the statistics from Ministry of Health of China in 2012, CHD accounts for 40.95% of all birth defects in China [3]. Given the large population, there may be a significant disease burden of CHD in China. So far, two meta-analyses of global birth prevalence of CHD have been published [2, 4]. Although these two articles provided a complete overview on the worldwide birth prevalence of CHD and its common subtypes from 1930 to 2017, and analyzed the prevalence of CHD in different continents, different income levels and different times, they did not separately analyze the CHD birth prevalence in China. Additionally, these reviews did not include all Chinese literatures because of language limitations, especially for these literatures published in Chinese journals, which may miss many important evidence of CHD birth prevalence in China. In fact, only a few studies assessing the birth prevalence of CHD among Chinese population are published in English.

Although the Chinese government began to monitor the birth prevalence of birth defects as early as 1986 [5], there has been not any study on the national CHD birth prevalence so far. Presently, China’ literatures on this topic are mainly local or single medical institution reports and only include the small sample size of study population, which causes that the findings are not comprehensive and cannot represent the national level [6, 7]. Overall, most of previous estimates on the birth prevalence of CHD in China have been based on point estimates from single studies [5]. China is a vast country with a vast territory, and the birth prevalence of CHD across provinces has not been accurately established, which may mask the influence of different medical diagnostic techniques, environment, heredity and social and economic inequalities on the birth prevalence of CHD between regions [8]. Especially, variation in CHD birth prevalence nationwide has been suggested [6, 9], but a complete overview is missing.

It is important to have reliable information about national CHD birth prevalence, because this can contribute to better understanding the impact of CHD on child health, providing the basic data for further research and prevention of CHD, and guiding future health resource allocation and advocacy efforts. In addition, dedicated care could be better planned and provided. Therefore, we performed a meta-analysis for the first time to provide a complete nationwide overview of the reported birth prevalence and spatial distribution of CHD and its specific subtypes in China from 1980 to 2019.

## Materials and methods

We reported this systematic review and meta-analysis according to the statement of the proposed preferred reporting items for systematic reviews and meta-analyses (PRISMA) [10].

### Search strategy

We systematically searched PubMed, Embase, Web of Science, China National Knowledge Infrastructure, Wanfang Database, Weipu and China Biology Medicine disc databases on May 31, 2019 to identify relevant studies assessing the birth prevalence of CHD among Chinese population. No time restriction for publication dates was used. We used and combined the following search terms: (congenital heart disease OR congenital heart defect OR congenital heart malformation OR congenital heart anomalies OR congenital cardiac disease OR congenital cardiac defect OR congenital cardiac malformation OR congenital cardiac anomalies OR congenital cardiovascular disease OR cardiovascular malformation OR cardiovascular defect OR cardiovascular anomalies OR birth defect OR congenital malformation OR congenital anomalies OR congenital defect) AND (prevalence OR incidence OR frequency OR epidemiology) AND (China OR Chinese). Terms covering “CHD”, “birth defect”, “prevalence” “incidence” and “China” were adjusted for each database. We reviewed the reference lists of all retrieved articles and recent reviews to reduce omissions.

### Outcomes of interest

In the present review, the outcomes of interest were CHD. The CHD diagnoses were identified according to the CHD codes of the International Statistical Classification of Diseases and Related Health Problems 10th Revision (ICD-10). We not only focused on the birth prevalence of total CHD, but also focused on the birth prevalence of specific subtypes of CHD. Total 21 commonly anatomical subtypes of CHD including atrial septal defect (ASD), ventricular septal defect (VSD), patent ductus arteriosus (PDA), tetralogy of Fallot (TOF), transposition of the great arteries (TGA), pulmonary stenosis (PS), atrioventricular septal defect, tricuspid atresia or stenosis, double outlet right ventricle, endocardial cushion defect, single ventricle, truncus arteriosus, hypoplastic left heart syndrome, pulmonary atresia, aortic stenosis, coarctation of the aorta, dextrocardia, ebstein anomaly, mitral insufficiency/regurgitation, total anomalous pulmonary venous return, and interrupted aortic arch were considered. Additionally, CHD could be also divided into mild lesions, severe lesions, cyanotic lesions and acyanotic lesions according to previous studies [11, 12] (details were shown in Supporting Information File 1).

According to China Birth Defect Monitoring Scheme (CBDMS) [13], birth defects were often monitored from 28 weeks of pregnancy in China. Presently, there are two monitoring models for the occurrence of birth defects in China including hospital-based monitoring model and population-based monitoring model. For hospital-based monitoring model, monitoring objects were perinatal infants born in the hospital from 28 weeks of gestation to 7 days after birth, including live births, fetal deaths or stillbirths and newborn deaths. The population-based monitoring model was carried out in communities, and the range of monitoring periods was from 28 weeks of gestation to 42 days after birth, including live births, fetal deaths or stillbirths and neonatal deaths. The mentioned-above two monitoring models were applied simultaneously in China. The birth prevalence of CHD was also monitored by two models. It should be noted that, so far, the monitoring objects of birth defects including CHD in China still do not cover all the populations. In the present review, we stipulated that the monitoring scheme provided by included studies must be consistent with CBDMS requirements.

### Selection criteria

We first performed an initial screening of titles or abstracts. A second screening was based on full-text review. Studies were considered eligible if they fulfilled the following criteria: (1) study participants were Chinese peoples; (2) studies published in Chinese or English language; (3) the reported monitoring scheme of CHD was consistent with CBDMS requirements; (4) the birth prevalence of CHD or its subtypes was reported (or data to calculate them); (5) the monitoring periods was from 28 weeks of gestation to 7 days or 42 days after birth; and (6) studies belonged to high quality studies after quality assessment. We excluded review papers, case–control studies, non-peer-reviewed local or government reports, conference abstract and presentation in the present review. We also excluded these studies that focused on the birth prevalence of CHD among specific populations, such as mothers with CHD, diabetes, hypertension, or other diseases that were significantly associated with the occurrence of CHD in offspring. If there were many studies based on the same research database, only the one that reported the most detailed data was included. Additionally, we assessed potential studies to ensure that there was no duplication of case series.

### Data extraction

Data extraction and quality assessment were done twice independently by two authors (ZLJ and QJB) to reduce the bias and errors of reviewers. Disagreements were resolved through discussions between investigators until a consensus was reached. The following study characteristics were recorded: publication year, geographic region, time period during which the study was performed, monitoring models of CHD (hospital-based monitoring model or population-based monitoring model), diagnostic method of CHD, number of monitoring participants, number of CHD cases, birth prevalence of total CHD and its specific subtypes, and quality score. Studies were grouped according to 5-year time periods since 1980 to demonstrate time trends. Time period is taken as the period in which the study was performed. Additionally, studies were further grouped according to geographic region [14] (northern region, southern region, eastern region, central region, western region and northeastern region) and income levels to estimate the birth prevalence of CHD in different regions and income groups. The income level groups were defined as: low income (≤ $1025), lower middle income ($1026 to $3995), upper middle income ($3996 to $12,375), and high income (≥ $12,376) according to World Bank Income groups [15].

### Quality assessment

We used the quality evaluation criteria for prevalence or incidence of a health problem proposed by Loney et al. [16] to assess the quality of included studies. The criterion sets 8 items to evaluate the literature from three aspects: the validity of the research method, the reasonable interpretation of the results, and the scope of application. The score ranges from 0 to 8 points, that is, the higher the score, the better the quality of the literature. When the study wins five or more scores, it is considered of higher methodological quality. In the present study, we excluded low quality studies.

### Statistical analysis

Statistical analyses were conducted using MS Excel, SPPS version 25, and Rmeta. ArcGIS 10.2 (ESRI, Redlands, CA) was applied for map construction. The combined birth prevalence and the corresponding 95% confidence intervals (CI) were calculated using either fixed-effect models or, in the presence of heterogeneity, random-effect models. Heterogeneity tests were performed using the Cochran Q test (*P* < 0.10 indicates statistically significant heterogeneity [17]) and *I*^2^ statistic.

Spatial autocorrelation analysis and Moran’s Index (I) were used to measure the patterns (cluster/disperse/random) of the birth prevalence of CHD throughout the country (Values for Moran’s I ranged from − 1 to + 1. The pattern of distribution was considered to be clustered if the value of Moran’s I was > 0 and *P* < 0.05; otherwise, the pattern was considered to be nonclustered [18]). Local spatial autocorrelation and the Getis-Ord Gi* statistic were used to identify the hot spots of CHD birth prevalence within the study area (the Gi* statistics is actually a Z score, and Z score values equal or greater than 1.96 were used to indicate the significance of observed hot spots with a *P* value of less than 0.05) [19, 20].

Time trends were plotted by using the Savitzky–Golay smoothing technique. Subgroup analysis was conducted based on gender (males and females), geographic region (northern region, southern region, eastern region, central region, western region and northeastern region; rural and urban areas), income levels (low income, lower middle income, upper middle income, and high income), and monitoring models (hospital-based monitoring model and population-based monitoring model), to estimate the birth prevalence of CHD in these subgroups. Pooled group estimates were compared with a Chi square test. Sensitivity analyses were further performed to examine the influence of various exclusion criteria on the overall risk estimate. Potential publication bias was assessed by Begg’s funnel plots and Egger’s linear regression test (significance level at *P* < 0.10). Statistical tests were declared significant for a two-tailed *P* value not exceeding 0.05, except where otherwise specified.

## Results

### Study selection and characteristics

**Fig. 1:**
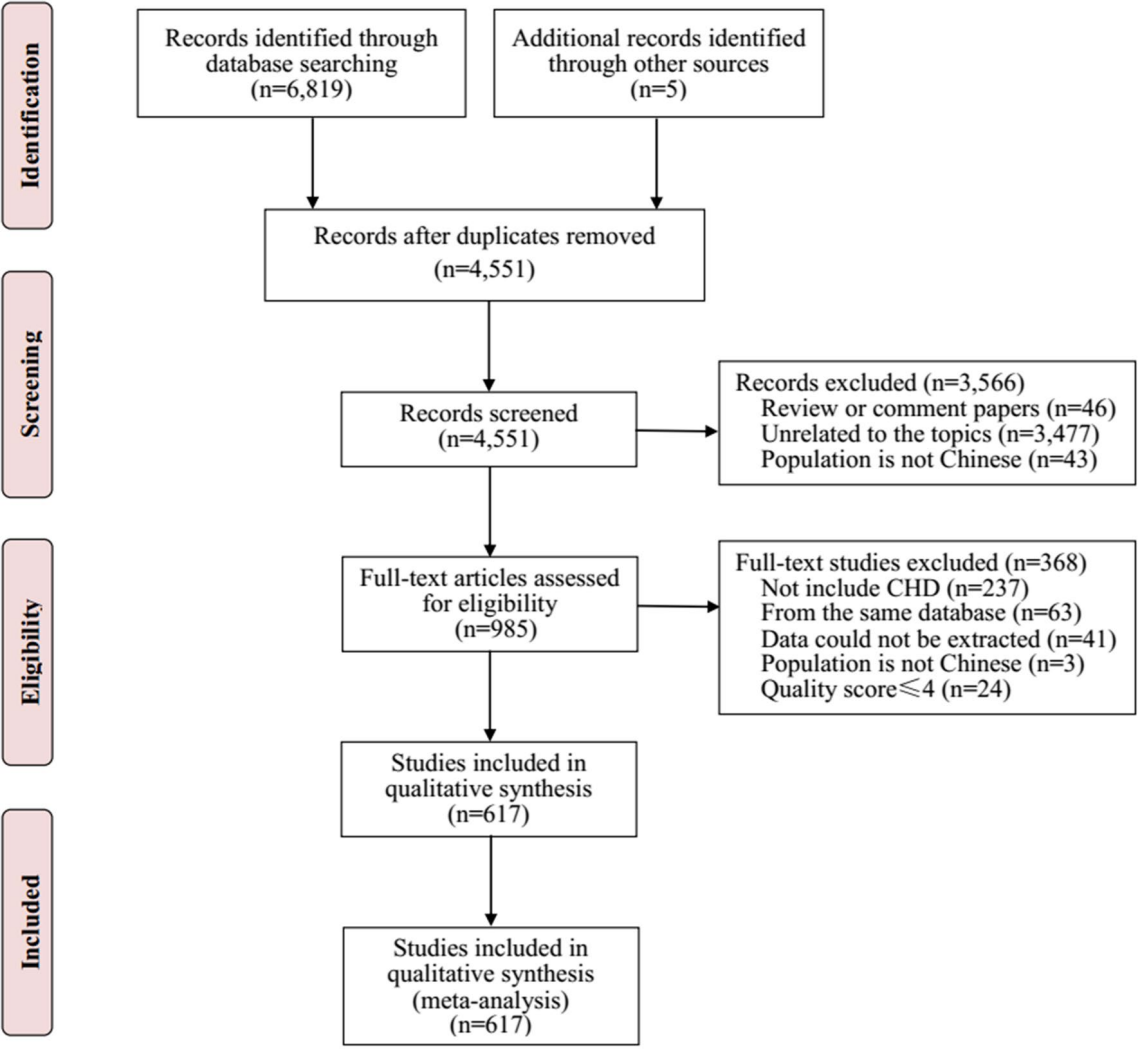
Flow chart of study identification and selection

The initial search returned a total of 6824 potential eligible publications from databases. Finally, total 617 studies involving 201,934 CHD individuals and 76,961,354 births were included for analysis (Fig. 1). The characteristics of included studies are summarized in Supplemental Excel. Among all included studies, more than half of studies (63.05%) were published after 2010. All included studies belonged to high quality studies, and monitored the occurrence of CHD according to the requirement of CBDMS. Most of studies (97.3%) used echocardiography as the main diagnostic tool; the remaining used combinations of diagnostic tools, such as death certificates, surgical reports, physical examination, and X-rays. The included studies involved in 32 provinces, autonomous regions or municipalities of China; of these, 325 studies (50.23%) were conducted in eastern region, 154 (23.80%) in western region, 385 (59.51%) in southern region, 260 (40.19%) in northern region, 116 (17.93%) in central region, and 50 (7.73%) in northeastern region.

### Birth prevalence of total CHD with time

**Fig. 2:**
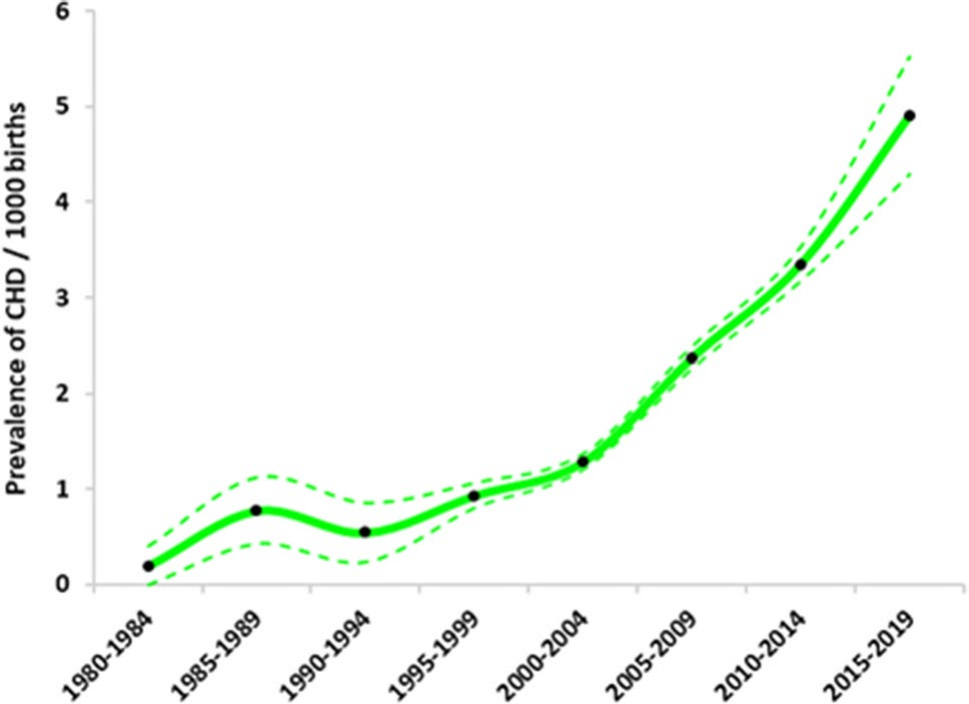
Total CHD birth prevalence over time in China. The solid line is the estimated birth prevalence, and dotted lines represent the 95% confidence interval

On average, the birth prevalence of total CHD in China during 1980–2019 was 2.502 (95% CI: 2.397, 2.607) per 1000 births. Reported total CHD birth prevalence increased substantially over time, from 0.201 per 1000 births (95% CI: 0.004, 0.398) in 1980–1984 to 4.905 per 1000 births (95% CI 4.288, 5.521) in 2015–2019. The birth prevalence of total CHD has been on the rise, except for a slight decline during the period 1990–1994. Overall, the increase trend over time of the birth prevalence of CHD was fold-line shaped, with a slow rise from 1980 to 2004 and a substantial rise from 2005 to 2019 (Fig. 2).

### Birth prevalence of total CHD among different genders

A total of 30 studies reported the birth prevalence of total CHD among different genders, involving 4,333,084 males (CHD identified in 14,910 individuals) and 3,758,334 females (CHD identified in 11,957 individuals). Reported birth prevalence of total CHD was 4.175 per 1000 births (95% CI: 3.524, 4.825) among males, and 3.533 per 1000 births (95% CI: 2.927, 4.140) among females. Overall, the birth prevalence of total CHD was significantly higher among male births compared with female births (χ^2^ = 40.955, *P *= 0.000).

Time trend analysis showed that total CHD birth prevalence increased substantially over time among both males and females (Supplemental Figure 1). Furthermore, total CHD birth prevalence among both males and females in eastern region, southern region and high-income provinces was significantly higher than that in other regions (Supplemental Figure 2).

### Birth prevalence of total CHD in urban and rural areas

Twenty-five studies reported the birth prevalence of total CHD in urban and rural areas, and totally 3,240,290 births (CHD identified in 13,375 individuals) in urban areas and 3,545,086 births (CHD identified in 8346 individuals) in rural areas were monitored. Reported birth prevalence of total CHD was 3.416 per 1000 births (95% CI: 2.547, 4.285) in urban area, and 2.582 per 1000 births (95% CI: 2.053, 3.111) in rural area. Overall, there was a statistically significant difference for the birth prevalence of total CHD between urban and rural areas (χ^2^ = 1668.676, *P *= 0.000).

Time trend analysis suggested that total CHD birth prevalence in urban area increased substantially from 1.759 per 1000 births (95% CI: 0.950, 2.568) in 1995–1999 to 4.547 per 1000 births (95% CI: 1.614, 7.479) in 2015–2019 (Supplemental Figure 3). However, total CHD birth prevalence in rural area was maintained at around 2.3 per 1000 births. Additionally, total CHD birth prevalence of both urban and rural areas in eastern region, southern region and high-income provinces were higher than that in other regions (Supplemental Figure 4).

### Birth prevalence of total CHD in different income areas

No data from lower-middle-income and low-income areas were available. Overall, there was statistically significant difference for total CHD birth prevalence in high-income areas [4.044 per 1000 births (95% CI: 3.788, 4.300)] compared with upper-middle-income areas [1.538 per 1000 births (95% CI: 1.441, 1.636)] (χ^2^ = 321.78, *P *= 0.000).

Time trend analysis showed, over time, total CHD birth prevalence in upper-middle-income areas increased substantially. However, total CHD birth prevalence in high-income areas significantly decreased from 1985 to 1994, increased substantially from 1995 to 2014, and started to lowly decrease from 2015 (Supplemental Figure 5).

### Birth prevalence of total CHD in different geographical regions

**Fig. 3:**
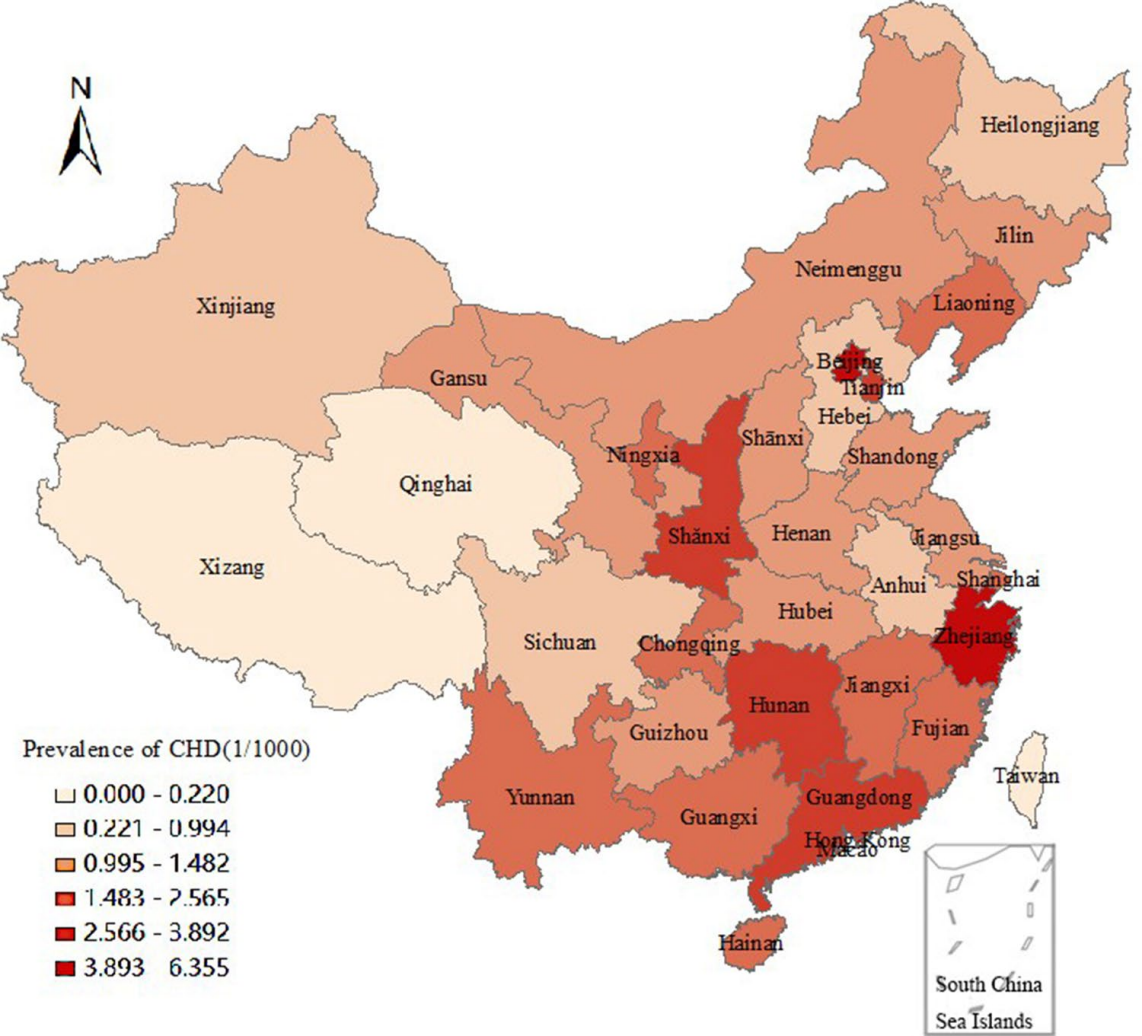
Geographical distribution of total CHD birth prevalence at the province level in China based on the average level during 1980–2019

**Fig. 4:**
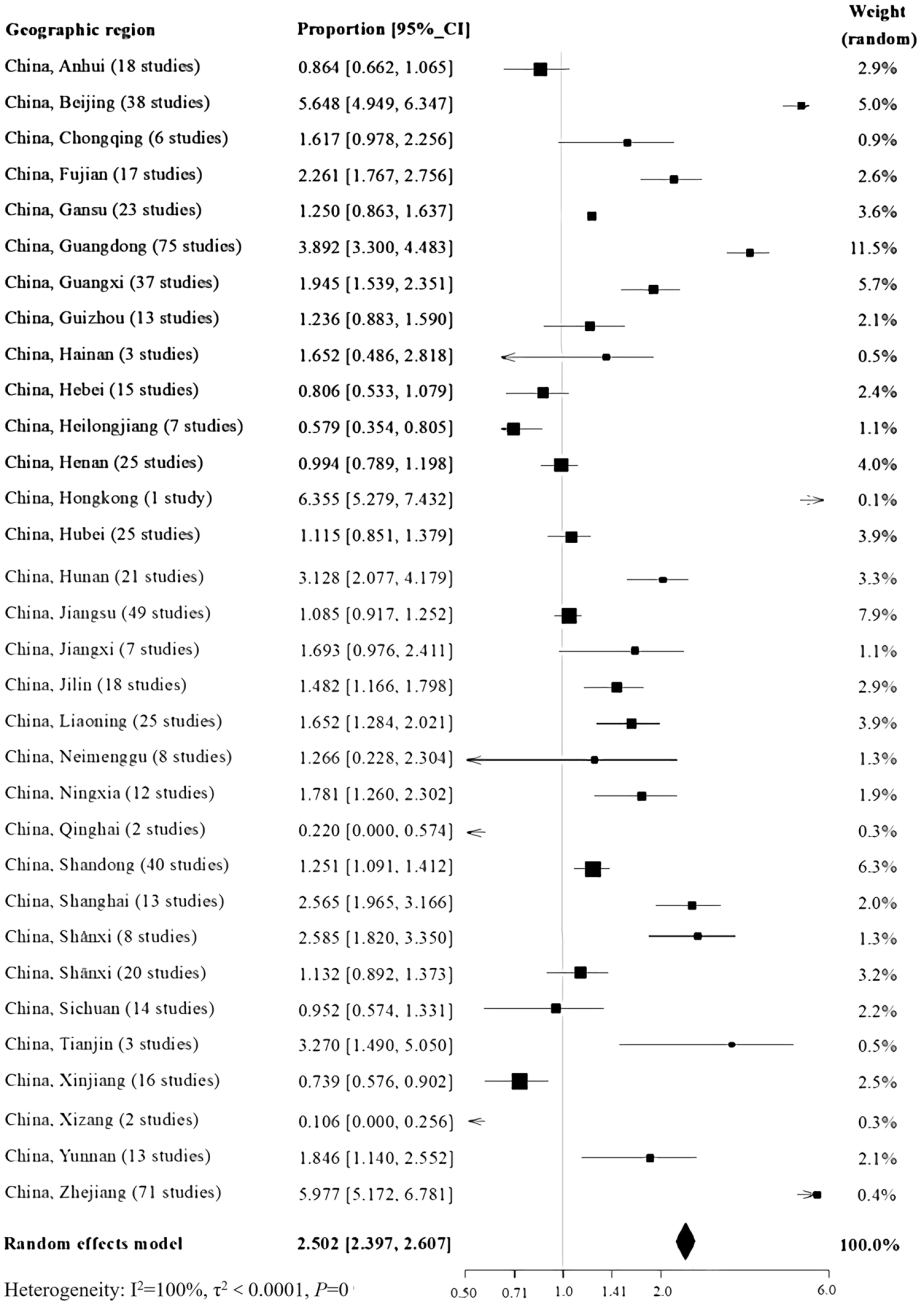
Total CHD birth prevalence per province in China

**Fig. 5:**
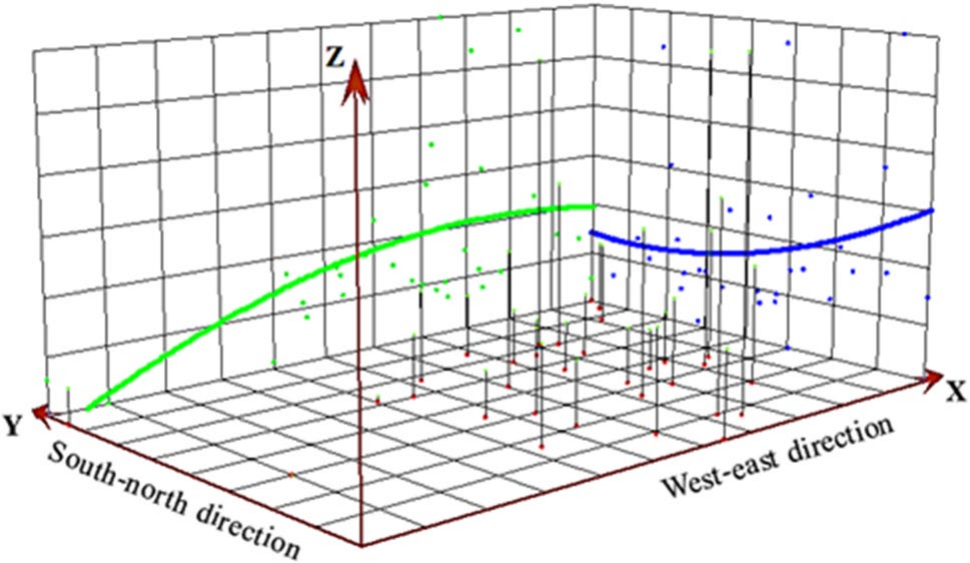
Three-dimensional trend analysis of total CHD birth prevalence in China

Significant geographical differences were found (Fig. 3). The highest reported total CHD birth prevalence was found in Hongkong [6.355 per 1000 births (95% CI: 5.279, 7.432)] and the lowest was found in Xizang [0.106 per 1000 births (95% CI: 0.000, 0.256)]. Total CHD birth prevalence of various provinces is shown in Fig. 4. The three-dimensional trend analysis showed that the birth prevalence of total CHD increased gradually from western region to eastern region, and decreased gradually from southern region to northern region (Fig. 5).

Global Moran’s I spatial autocorrelation analysis (Supplemental Figure 6) showed that the birth prevalence of total CHD in different provinces didn’t have spatial clustering (Moran’s I values was − 0.154, *P *= 0.115); additionally, local spatial autocorrelation analysis (Supplemental Figure 7) also indicated that there was no aggregation among 32 provinces (Z = 1.987, *P *= 0.124). Time trend analysis showed that total CHD birth prevalence in different geographical regions (eastern, western, northern, southern, central, and northeastern regions) increased substantially over time (Supplemental Figure 8 and 9).

### Birth prevalence of total CHD among different monitoring models

Of the 617 studies included in this review, fifty studies (8.1%) adopted population-based monitoring model to monitor the occurrence of CHD; the remaining studies (91.9%) adopted hospital-based monitoring model. Overall, total CHD birth prevalence was significantly higher among population-based monitoring model [3.373 per 1000 births (95% CI: 2.897, 3.848)] compared with hospital-based monitoring model [2.441 per 1000 births (95% CI: 2.332, 2.549)]. Time trend analysis showed that total CHD birth prevalence among different monitoring models increased substantially over time (Supplemental Figure 10).

### Birth prevalence of specific subtypes of CHD

**Fig. 6:**
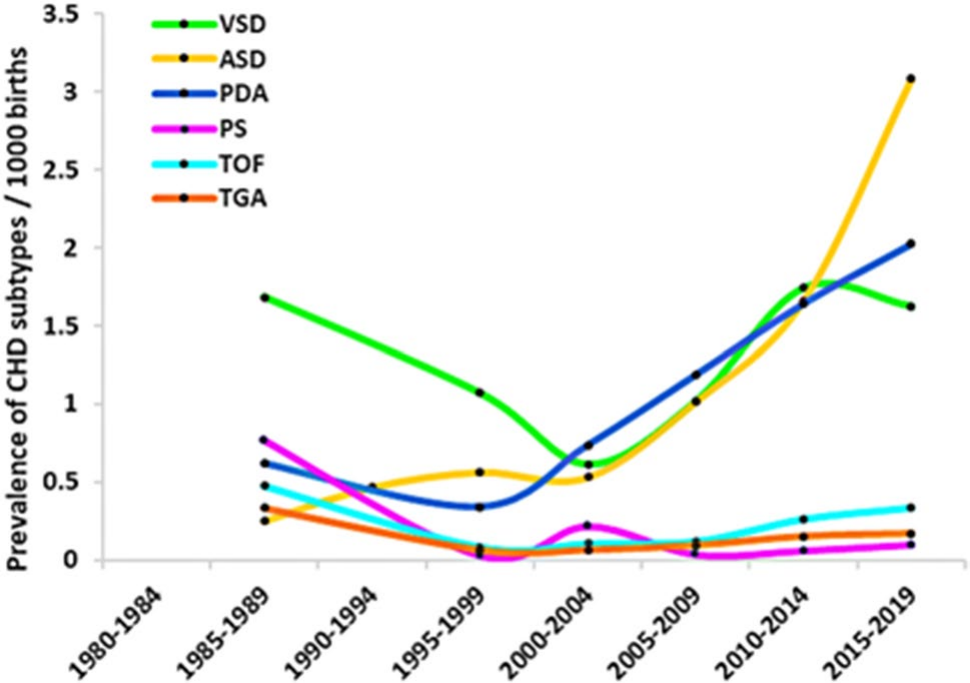
Birth prevalence of six specific subtypes of CHD over time in China

The birth prevalence of specific subtypes of CHD is summarized in Table 1. Overall, VSD [1.410 per 1000 births (95% CI: 1.176, 1.645)], ASD [1.314 per 1000 births (95% CI: 0.994, 1.635)], PDA [1.294 per 1000 births (95% CI: 1.037, 1.550)], TOF [0.210 per 1000 births (95% CI: 0.151, 0.268)], TGA [0.133 per 1000 births (95% CI: 0.092, 0.175)], Tricuspid atresia or stenosis [0.125 per 1000 births (95% CI: 0.036, 0.213)], and PS [0.066 per 1000 births (95% CI: 0.036, 0.096)] were identified as the first seven most frequent subtypes and they contributed 74.3% to the total burden of CHD. Time trend analysis showed that the birth prevalence of ASD and PDA increased substantially over time; the birth prevalence of VSD slowly decreased from 1985 to 2004, increased substantially from 2005 to 2014, and slowly decreased from 2015; the remaining subtypes remained a stable status over time (Fig. 6) (of note, time trend analysis was performed only for six common subtypes because of limited information provided by included studies).

**Table 1:** The birth prevalence and percentage of specific CHD subtypes

Specific subtypes	Number of studies	Event	Total	Birth prevalence, ‰ (95% confidence interval)	Percentage, % (95% confidence interval)
Twenty-one specific subtypes of CHD					
Atrial septal defect	22	4967	3,750,686	1.314 (0.994–1.635)	27.365 (26.716–28.013)
Patent ductus arteriosus	21	3750	3,748,546	1.294 (1.037–1.550)	20.660 (20.071–21.249)
Ventricular septal defect	22	3,397	3,750,686	1.410 (1.176–1.645)	18.715 (18.148–19.283)
Tetralogy of fallot	19	661	3,466,248	0.210 (0.151–0.268)	3.642 (3.369–3.914)
Atrioventricular septal defect	14	488	3,394,004	0.146 (0.101–0.190)	2.689 (2.453–2.924)
Tricuspid atresia or stenosis	8	322	1,917,479	0.125 (0.036–0.213)	1.774 (1.582–1.966)
Transposition of the great arteries	14	268	2,289,051	0.133 (0.092–0.175)	1.477 (1.301–1.652)
Pulmonary stenosis	12	159	2,325,983	0.066 (0.036–0.096)	0.876 (0.740–1.012)
Double outlet right ventricle	8	122	2,122,417	0.059 (0.028–0.090)	0.672 (0.553–0.791)
Endocardial cushion defect	11	74	1,779,790	0.048 (0.025–0.071)	0.408 (0.315–0.500)
Single ventricle	12	64	1,858,797	0.034 (0.018–0.051)	0.353 (0.266–0.439)
Truncus arteriosus	9	47	1,533,718	0.038 (0.014–0.062)	0.259 (0.185–0.333)
Hypoplastic left heart syndrome	7	35	1,464,418	0.021 (0.007–0.035)	0.193 (0.129–0.257)
Pulmonary atresia	7	31	1,302,023	0.028 (0.005–0.052)	0.171 (0.111–0.231)
Aortic stenosis	5	24	1,214,226	0.016 (0.009–0.024)	0.132 (0.079–0.185)
Coarctation of the aorta	5	19	516,806	0.035 (0.019–0.051)	0.105 (0.058–0.152)
Dextrocardia	4	9	130,700	0.054 (0.014–0.094)	0.050 (0.017–0.082)
Ebstein anomaly	3	8	989,085	0.007 (0.002–0.012)	0.044 (0.014–0.075)
Mitral insufficiency/regurgitation	1	3	29,920	0.100 (0.000–0.214)	0.017 (0.000–0.035)
Total anomalous pulmonary venous return	1	2	20,928	0.096 (0.000–0.228)	0.011 (0.000–0.026)
Interrupted aortic arch	0	0	0	–	–
Four lesions of CHD					
Severe lesions	21	2134	3,748,546	0.762 (0.611–0.913)	11.757 (11.288–12.226)
Mild lesions	22	12,316	3,750,686	4.581 (3.755–5.407)	67.853 (67.174–68.533)
Cyanotic lesions	20	1719	3,509,203	0.608 (0.465–0.751)	9.471 (9.045–9.897)
Acyanotic lesions	22	12,731	3,750,686	4.781 (3.934–5.627)	70.139 (69.474–70.805)
Total CHD	617	201,934	76,961,354	2.502 (2.397–2.607)	–

*CHD* congenital heart disease

**Fig. 7:**
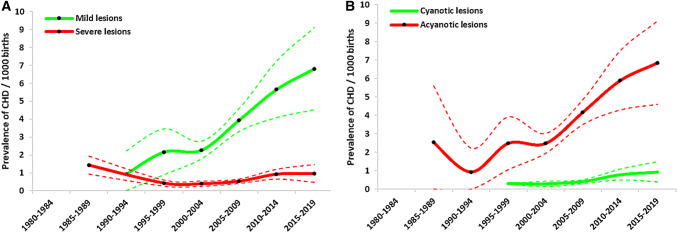
Birth prevalence of mild lesions, severe lesions, cyanotic lesions, and acyanotic lesions over time in China. The solid line is the estimated birth prevalence, and dotted lines represent the 95% confidence interval

Our study also showed that the birth prevalence of mild lesions [4.581 per 1000 births (95% CI: 3.755, 5.407)] was significantly higher than severe lesions [0.762 per 1000 births (95% CI: 0.611, 0.913)]; similarly, the birth prevalence of acyanotic lesions [4.781 per 1000 births (95% CI: 3.934, 5.627)] was also significantly higher than cyanotic lesions [0.608 per 1000 births (95% CI: 0.465, 0.751)]. Time trend analysis suggested that the birth prevalence of mild lesions and acyanotic lesions significantly increased over time, but the birth prevalence of severe lesions and cyanotic lesions remained a stable status over time (Fig. 7).

### Heterogeneity, subgroup analyses, and publication bias

Significant heterogeneity was observed within pooled estimates for gender, geographic region, monitoring models and income levels (*I*^2^ range 91.2–100%; all *P* < 0.001) (Supplemental Table 1). Birth prevalence estimates did not change significantly after exclusion of any single study. However, both the funnel plot (Supplemental Figure 11) and egger’s regression test (*P* < 0.01) indicated the evidence of publication bias.

## Discussion

In the present review, we firstly quantified the birth prevalence of both CHD and its specific subtypes in China by using data from 617 published studies involving 201,934 CHD individuals and 76,961,354 births. Our study yielded several main findings. First, we found that total CHD birth prevalence increased significantly over time in China, from 0.201 per 1000 births in 1980–1984 to 4.905 per 1000 births in 2015–2019, with an increase of 24 times. Second, for total CHD birth prevalence in China, significantly differences across gender, geographical regions, income levels, and monitoring models were observed. Third, total CHD birth prevalence increased gradually from western region to eastern region, and decreased gradually from southern region to northern region in China, without aggregation characteristics among 32 provinces. Fourth, in China, the reported CHD was dominated by mild lesions (67.8%) and acyanotic lesions (70.1%). Additionally, VSD, ASD, PDA, TOF, TGA, tricuspid atresia or stenosis and PS were identified as the first seven most frequent subtypes.

Obviously, total CHD birth prevalence in China (2.502‰) from 1980 to 2019 was relatively lower compared with both the worldwide prevalence (8.2‰) and the Asia prevalence (9.3‰) reported by previous two studies [2, 4]. We speculated that there may be several reasons for this difference. First, this phenomenon might be attributed to the narrow diagnosis window. In the previous two studies [2, 4] assessing the birth prevalence of CHD, they monitored the occurrence of CHD until 5 to 6 years after birth. However, compared with these studies, our monitoring period was significantly shorter (7 or 42 days after birth), which makes it difficult to identify more CHD cases after discharge [21]. Additionally, some developed countries started to monitor the occurrence of CHD from 20 weeks of gestation, but we started from 28 weeks of gestation, which may also bring about an underestimation of total CHD birth prevalence in the present review. For example, previous studies confirmed that the birth prevalence of CHD monitored from 20 weeks of pregnancy was five times as high as that monitored from 28 weeks of pregnancy [22]. Second, although the Chinese government began monitoring birth defects in 1986 [5], data related to CHD were not available in many areas of China until 1998 due to a lack of echocardiographic techniques [23]. Nowadays, echocardiography is not available in many grassroots delivery facilities, and the data for newborns with suspected CHD who were transferred to another medical center for diagnosis were not documented by the surveillance system [24]. As a result, many actually diagnosed CHD children were not recorded, which may cause the underestimation of present results.

Third, presently, many countries adopted population-based monitoring model to monitor the prevalence of CHD [11], but China mainly adopted hospital-based monitoring model. In China, the population-based monitoring model remains at its early stage and has not been comprehensively extended. In the present study, we found the birth prevalence of CHD from population-based monitoring model was significantly higher that from hospital-based monitoring model. Furthermore, the detection rate of CHD can be greatly affected by variations in definitions, selection criteria, diagnostic methods and skills of physicians across different participating countries and hospitals [25]. Given mentioned-above factors, it may be understandable that the birth prevalence of CHD in present study was obviously lower than the two previous meta-analyses. Here, we suggest that the Chinese government should comprehensively promote the population-based monitoring model of birth defects, and should rationally allocate health resources to improve the diagnostic ability of CHD, especially in economically underdeveloped areas. In addition, monitoring of CHD should be integrated into pediatric cardiac centers or institutions to increase the detection rate of later-presenting CHD individuals and finally obtain more comprehensive epidemiological data.

Presently, the reasons for the substantial increase of reported CHD birth prevalence in China are still uncertain. A universal viewpoint is that improved diagnostic and screening methods themselves, parental lifestyles, genetic and environmental factors associated birth defects, or a combination of these factors, bring about increased birth prevalence of CHD. Increase of CHD birth prevalence may be a result of widely used of echocardiography in clinical practice [26], making it possible to diagnose patients with mild lesions and asymptomatic patients. Meantime, echocardiography currently is often used as a screening tool before (noncardiac) surgery or full assessment in case of noncardiac disease, causing an increase in diagnoses of minor lesions such as a small VSD or ASD [2].

An additional possibility is that the changes in environmental exposures over time may be associated with substantial increase of CHD. For example, it has reported that the industrialization and urbanization over the past century have had effects on CHD birth prevalence [27]. Nowadays, more and more data of parental lifestyle during pregnancy, such as parental smoking, alcohol consumption, maternal pre-gestational illness, various therapeutic drug exposures, exposure to organic solvents and other environmental teratogen have been proven to be associated with increased risk of CHD [27, 28]. However, until now, it remains uncertain whether detected differences represent true or merely methodological differences. In sharp contrast with the worldwide CHD birth prevalence that had a relative stabilization in 1995–2009 [4], we found the total CHD birth prevalence in China continues to rise during this time. This may be because echocardiography has been widely applied and fully popularized in China during this period, increasing the detection rate of CHD and masking its actual birth prevalence.

In our review, the important geographical differences for CHD birth prevalence were found. The southern region and eastern region in China experienced higher birth prevalence for total CHD compared with other regions. The total birth prevalence rate of the national CHD gradually increased from the western region to the eastern region, and gradually decreased from the southern region to the northern region, which was positively related to the economic development. However, a study conducted at the Qinghai Women and Children Hospital in Xining indicated that the birth prevalence of CHD at high altitude areas was significantly higher than that at low altitude regions [29]. There are some high altitude areas in some provinces in western China (e.g. Xinjiang, Xizang and Qinghai), but the total CHD birth prevalence in these provinces are relatively low. We speculated that this difference can be attributed in part to the different popularity of echocardiography and differences in healthcare and referral systems. Furthermore, economic underdevelopment, reduced pollution factories and reduced environmental pollution may also be important reasons for the low incidence of CHD in western and northern China.

Present study showed that the CHD birth prevalence in urban areas was higher than that in rural areas, which contradicts the report by Li Dai et al. [5]. The phenomenon is explained to some extent by the stronger overall health awareness, the wider use of prenatal diagnosis techniques, more accessibility and reporting practices in urban areas. In addition, we found significant differences in the birth prevalence of CHD based on income levels, which also favored that a lack of resources, medical insurance, screening programs, and referral systems could lead to underestimation of true birth prevalence. Hence, the future CHD birth prevalence studies should try to adjust the impact of socioeconomic factors to determine the true level of CHD prevalence and other influencing factors. Meantime, we recommended that some policies to address the epidemiology of CHD in deprived areas of western China and northern China should be done, especially, medical resources should be better allocated, and local training programs should be also developed, finally to avoid missing more CHD cases in these areas.

Important population differences were found. Reported birth prevalence of CHD in male births was higher than that in female births, which is consistent with some previous studies [29, 30]. The exact mechanisms involved in the higher CHD birth prevalence in males are still unclear and warrant further research. A possible hypothesis in a recent population-based study in the United Kingdom reported that this difference may be explained by the higher susceptibility of Y chromosome than X chromosome [31]. We also found the birth prevalence of CHD was significantly higher in population-based monitoring model versus hospital-based monitoring model, which could be attributed to the longer monitoring period among the population-based monitoring model compared with the hospital-based monitoring model. By 2010, 90% of the countries worldwide had used the population-based monitoring model for the monitoring of birth defects [29]. However, the population-based monitoring model in China is only at its beginning stages, covering only one-sixth of populations that hospital-based monitoring model does [5]. To meet the need of prevention of birth defects, measures should be taken to improve the national and provincial birth defects surveillance systems in China.

This study has some limitations. First, although we investigated all available literatures of CHD birth prevalence in China, and checked for bias caused by regions, surveillance models and income levels, some residual bias may be present in our estimates. It remains uncertain whether the difference in the detected birth prevalence of CHD presents real or just methodological. Second, when assessing the birth prevalence and time-trends of specific CHD subtypes, we only relied on a small quantity of studies (22 studies). Meantime, the application of different versions of ICD codes in included studies (ICD-9, ICD-10 and Maternal and Child Health Monitoring Manual in China) may have a weak influence on the recording of CHD subtypes. Also, discrepancies in antenatal or postnatal diagnosis of birth defects between surveillance institutes may affect the detection rate and introduce potential biases.

Third, the heterogeneity in our study was substantial. Although subgroup analyses have been conducted according to gender, geographic regions, income level and surveillance models, significant evidence of heterogeneity was still observed among subgroups. However, the heterogeneity was expected and inevitable considering the differences in the obstetric management, diagnosis of CHD, length of follow up, ethnic background, socioeconomic situation, study population, food and life habits, prenatal care services, and referral systems. Fourth, because some studies did not provide annual data on the birth prevalence of CHD, we adopted this way that a same study (e.g., performed from 2003 to 2007) could be counted twice (2000–2004 and 2005–2009) when performing the time trend analysis, which may have a slight impact on the results of time trend analysis. Furthermore, potential publication bias could influence the findings. In this study, both the funnel plot and egger’s regression test indicated the evidence of publication bias. The asymmetry of funnel plots may be due to small-study effects, especially when there was evidence of substantial heterogeneity across studies [32]. Besides, this meta-analysis reviewed only published studies, conference abstracts, case–control studies and non-peer-reviewed local or government reports were excluded, making it susceptible to the effect of publication bias. Compared to the studies clustered around the central estimates with low standard errors, most of the studies that are located far right of the funnel plot were small sample studies, and almost half of them were studied in high prevalence provinces (e.g. Zhejiang province and Beijing province), making it more easily to be published. Another inevitable limitation of present study was that it does not really cover the entire Chinese population. Data from many provinces (e.g. Qinghai, Xizang and Hainan) were sparse and unrepresentative. In the future, birth defect registration covering the entire Chinese population and adjusting the potential environmental influences on CHD are needed to determine the exact birth prevalence.

## Conclusions

Total CHD birth prevalence in China increases continuously in the past forty years. Significant differences in gender, geographical regions, income levels, and monitoring models were found for birth prevalence of CHD. It remains uncertain whether detected differences in birth prevalence of CHD represent true or merely methodological differences. In the future, population wide prospective birth defect registries covering the entire Chinese population need to determine the exact birth prevalence.

## Electronic supplementary material

Below is the link to the electronic supplementary material.

Supplemental Figure 1: Total CHD birth prevalence over time in different genders in China. The solid line is the estimated birth prevalence, and dotted lines represent the 95% confidence interval. (PNG 34 kb)

Supplemental Figure 2: Geographical distribution of total CHD birth prevalence in different genders. (PNG 192 kb)

Supplemental Figure 3: Total CHD birth prevalence over time in urban and rural areas in China. The solid line is the estimated birth prevalence, and dotted lines represent the 95% confidence interval. (PNG 31 kb)

Supplemental Figure 4: Geographical distribution of total CHD birth prevalence in urban and rural areas. (PNG 190 kb)

Supplemental Figure 5: Total CHD birth prevalence over time in different income levels in China. The solid line is the estimated birth prevalence, and dotted lines represent the 95% confidence interval. (PNG 40 kb)

Supplemental Figure 6: Global Moran’s I spatial autocorrelation analysis of total CHD birth prevalence in China. (PNG 186 kb)

Supplemental Figure 7: Local spatial autocorrelation analysis of total CHD birth prevalence in China. (JPEG 233 kb)

Supplemental Figure 8: Total CHD birth prevalence over time in different geographic regions (south-north direction) in China. (PNG 44 kb)

Supplemental Figure 9: Total CHD birth prevalence over time in different geographic regions (west-east direction) in China. The solid line is the estimated birth prevalence, and dotted lines represent the 95% confidence interval. (PNG 38 kb)

Supplemental Figure 10: Total CHD birth prevalence over time in different monitoring models in China. The solid line is the estimated birth prevalence, and dotted lines represent the 95% confidence interval. (PNG 41 kb)

Supplemental Figure 11: Funnel plots with 95% confidence limits of included studies. (PNG 50 kb)

Supplemental Table 1 (DOCX 18 kb)

Supporting information file 1 (DOCX 13 kb)

Supplemental Excel: The characteristics of the included studies. (XLSX 239 kb)
