# Supplementary figures and images for: Birth prevalence of congenital heart disease in China, 1980–2019: a systematic review and meta-analysis of 617 studies
Source: Eur J Epidemiol. 2020 Jun 9;35(7):631–42. doi: 10.1007/s10654-020-00653-0 (PMC7387380; doi:10.1007/s10654-020-00653-0)

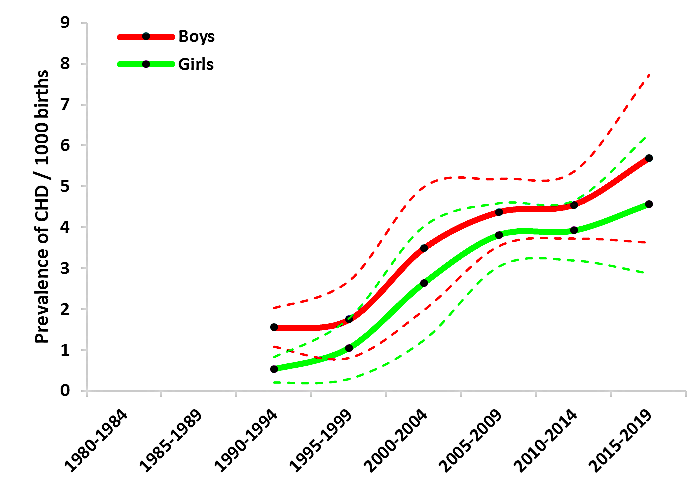

Supplement: Supplementary file 1 — Supplemental Figure 1: Total CHD birth prevalence over time in different genders in China. The solid line is the estimated birth prevalence, and dotted lines represent the 95% confidence interval. (PNG 34 kb) [file 10654_2020_653_MOESM1_ESM.png]

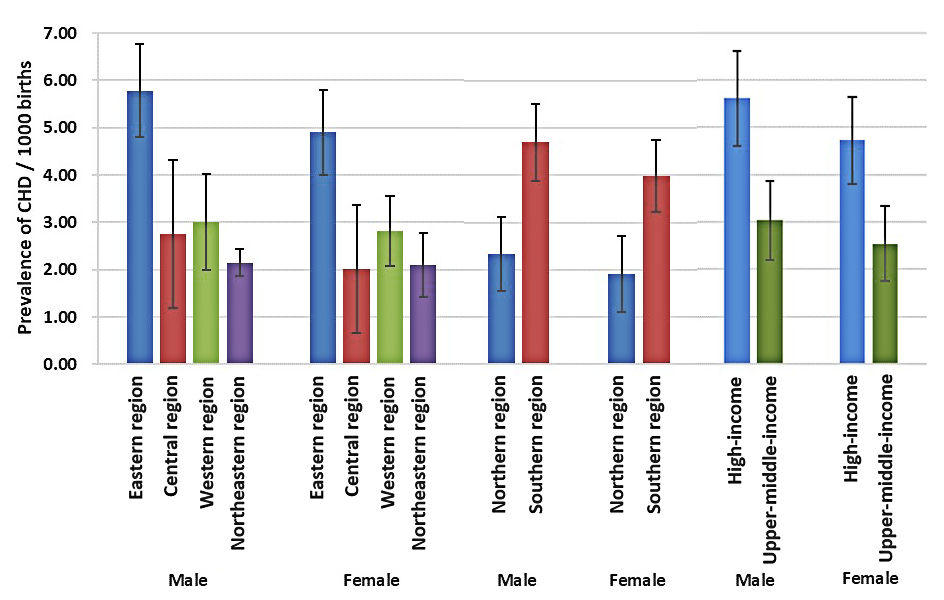

Supplement: Supplementary file 2 — Supplemental Figure 2: Geographical distribution of total CHD birth prevalence in different genders. (PNG 192 kb) [file 10654_2020_653_MOESM2_ESM.png]

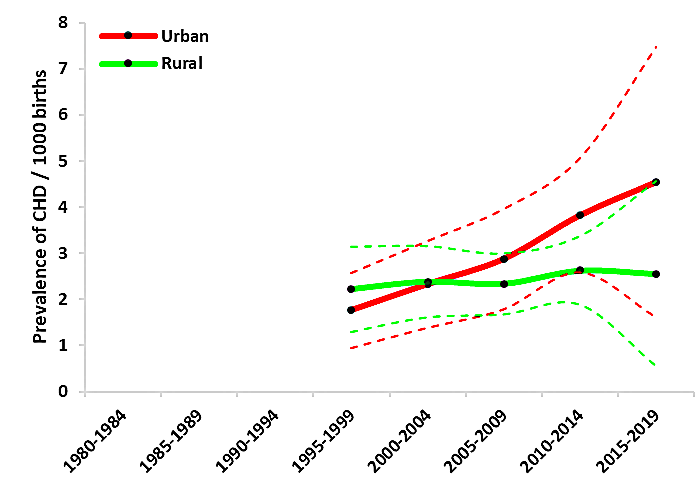

Supplement: Supplementary file 3 — Supplemental Figure 3: Total CHD birth prevalence over time in urban and rural areas in China. The solid line is the estimated birth prevalence, and dotted lines represent the 95% confidence interval. (PNG 31 kb) [file 10654_2020_653_MOESM3_ESM.png]

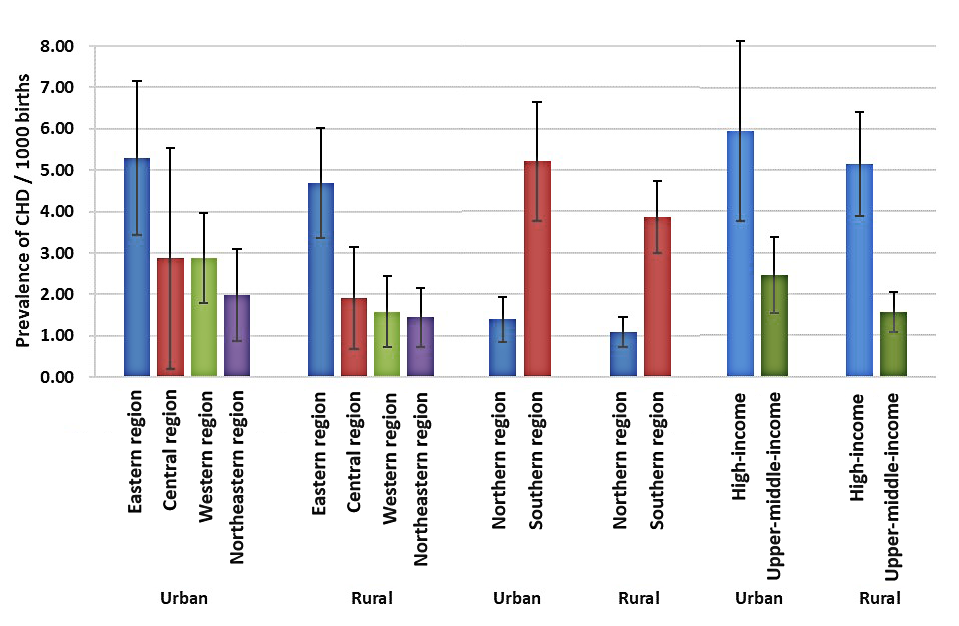

Supplement: Supplementary file 4 — Supplemental Figure 4: Geographical distribution of total CHD birth prevalence in urban and rural areas. (PNG 190 kb) [file 10654_2020_653_MOESM4_ESM.png]

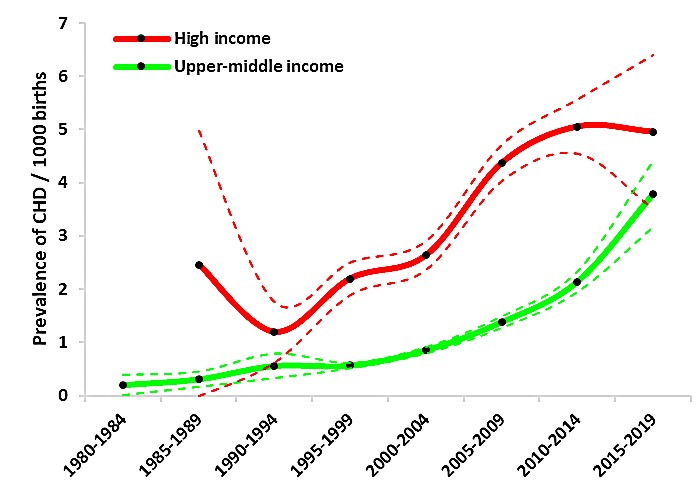

Supplement: Supplementary file 5 — Supplemental Figure 5: Total CHD birth prevalence over time in different income levels in China. The solid line is the estimated birth prevalence, and dotted lines represent the 95% confidence interval. (PNG 40 kb) [file 10654_2020_653_MOESM5_ESM.png]

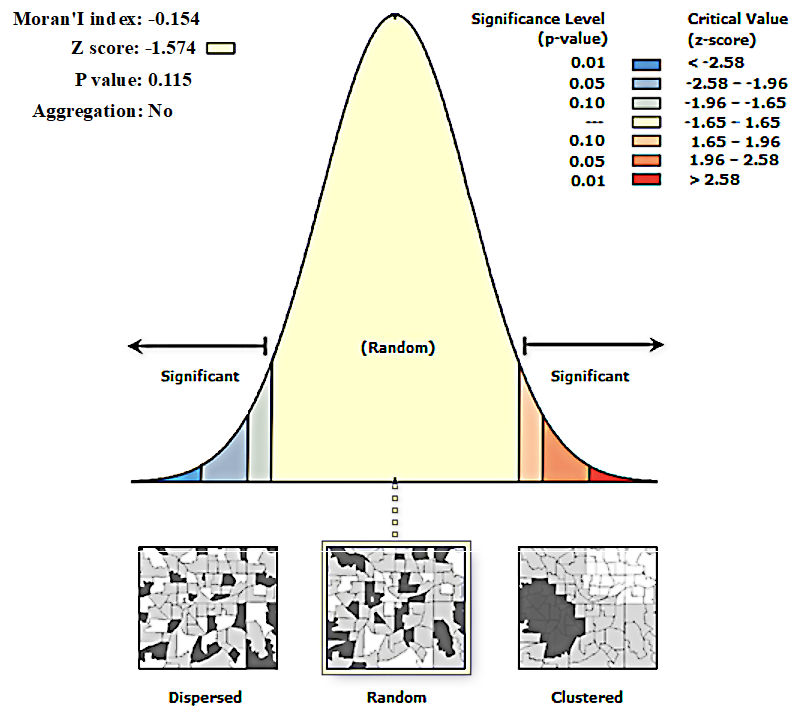

Supplement: Supplementary file 6 — Supplemental Figure 6: Global Moran’s I spatial autocorrelation analysis of total CHD birth prevalence in China. (PNG 186 kb) [file 10654_2020_653_MOESM6_ESM.png]

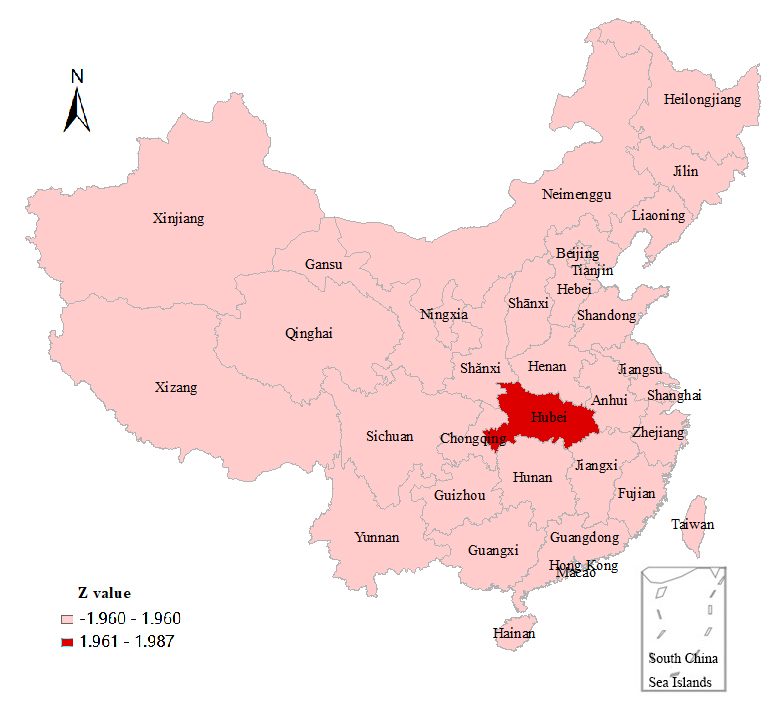

Supplement: Supplementary file 7 — Supplemental Figure 7: Local spatial autocorrelation analysis of total CHD birth prevalence in China. (JPEG 233 kb) [file 10654_2020_653_MOESM7_ESM.jpg]

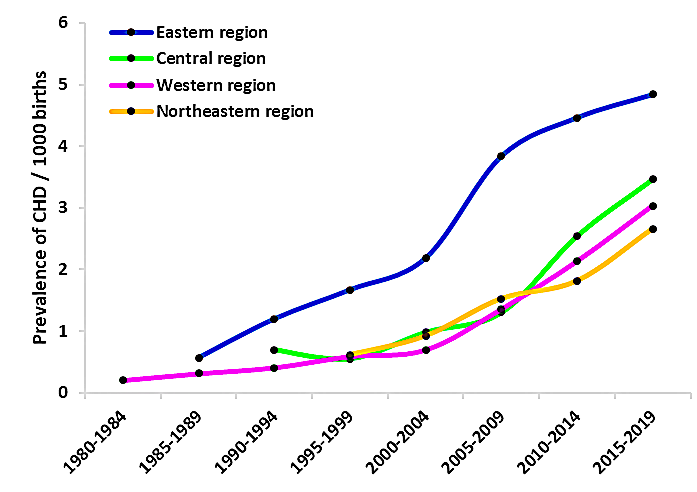

Supplement: Supplementary file 8 — Supplemental Figure 8: Total CHD birth prevalence over time in different geographic regions (south-north direction) in China. (PNG 44 kb) [file 10654_2020_653_MOESM8_ESM.png]

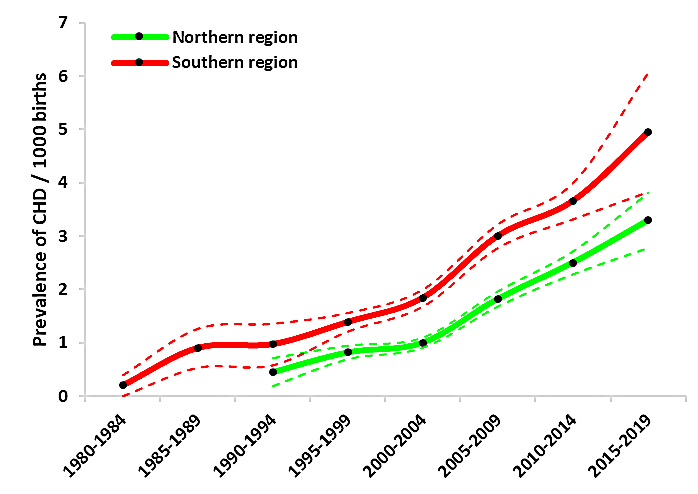

Supplement: Supplementary file 9 — Supplemental Figure 9: Total CHD birth prevalence over time in different geographic regions (west-east direction) in China. The solid line is the estimated birth prevalence, and dotted lines represent the 95% confidence interval. (PNG 38 kb) [file 10654_2020_653_MOESM9_ESM.png]

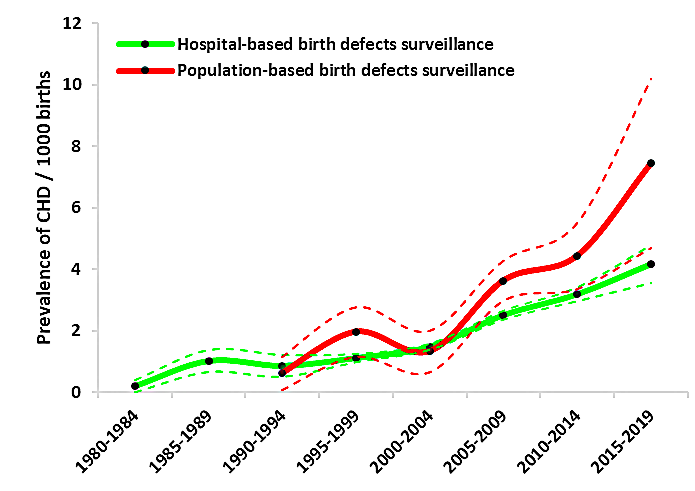

Supplement: Supplementary file 10 — Supplemental Figure 10: Total CHD birth prevalence over time in different monitoring models in China. The solid line is the estimated birth prevalence, and dotted lines represent the 95% confidence interval. (PNG 41 kb) [file 10654_2020_653_MOESM10_ESM.png]

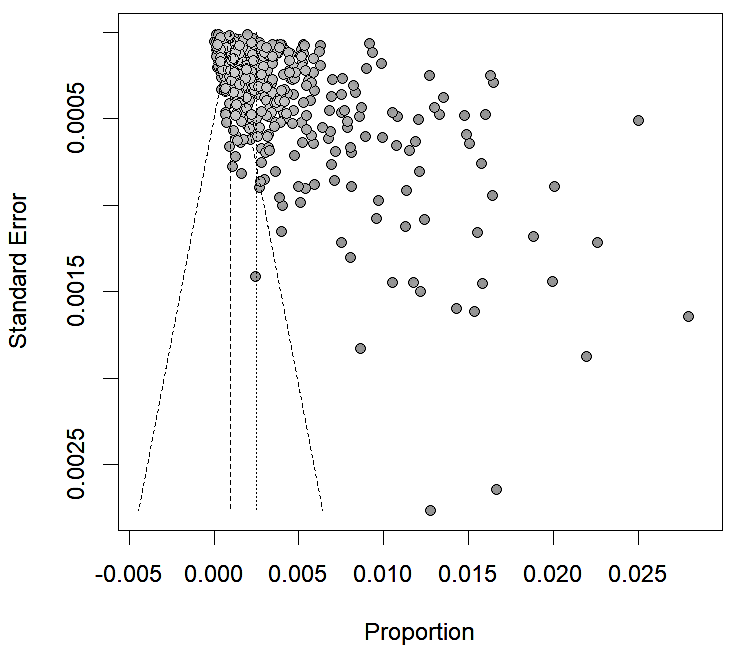

Supplement: Supplementary file 11 — Supplemental Figure 11: Funnel plots with 95% confidence limits of included studies. (PNG 50 kb) [file 10654_2020_653_MOESM11_ESM.png]
