# Supplementary material for: Birth prevalence of congenital heart disease in China, 1980–2019: a systematic review and meta-analysis of 617 studies
Source: Eur J Epidemiol. 2020 Jun 9;35(7):631–42. doi: 10.1007/s10654-020-00653-0 (PMC7387380; doi:10.1007/s10654-020-00653-0)
Supplement: Supplementary file 12 — Supplemental Table 1 (DOCX 18 kb) [file 10654_2020_653_MOESM12_ESM.docx]

**Supplemental Table 1: Subgroup analysis for total CHD birth prevalence in China.**

| **Subgroup variables (TSD)** | **Number**  **of Studies** | **Event** | **Total** | **Birth prevalence, ‰ (95% CI)** |
| --- | --- | --- | --- | --- |
| **Area 1 (*χ*^2^=298.96, *P*=0.000）** | |  |  |  |
| Eastern region | 325 | 117,863 | 32,231,836 | 3.434 (3.240-3.628) |
|  |  |  |  | χ^2^=81106.05, *P*=0.000; I^2^=99.6% |
| Central region | 116 | 25,615 | 12,470,267 | 1.577 (1.398-1.757) |
|  |  |  |  | χ^2^=18945.63, *P*=0.000; I^2^=99.4% |
| Western region | 154 | 32,684 | 17,607,198 | 1.488 (1.334-1.642) |
|  |  |  |  | χ^2^=17239.39, *P*=0.000; I^2^=99.1% |
| Northeastern region | 50 | 8,144 | 5,613,505 | 1.435 (1.227-1.644) |
|  |  |  |  | χ^2^=2818.29, *P*=0.000; I^2^=98.3% |
| NA | 2 | 17,628 | 9,038,548 | 2.320 (1.521-3.118) |
|  |  |  |  | χ^2^=11.38, *P*=0.000; I^2^=91.2% |
| **Area 2 (*χ*^2^=141.32, *P*=0.000）** | |  |  |  |
| Northern region | 260 | 45,721 | 28,118,093 | 1.682 (1.576-1.789) |
|  |  |  |  | χ^2^=25,071.63, *P*=0.000; I^2^=99.0% |
| Southern region | 385 | 138,585 | 39,804,713 | 2.871 (2.706-3.036) |
|  |  |  |  | χ^2^=98092.42, *P*=0.000; I^2^=99.6% |
| Not stated | 2 | 17,628 | 9,038,548 | 2.320 (1.521-3.118) |
|  |  |  |  | χ^2^=11.38, *P*=0.000; I^2^=91.2% |
| **Income levels (*χ*^2^=321.78, *P*=0.000）** | | |  |  |
| High-income | 260 | 111,292 | 26,203,401 | 4.044 (3.788-4.300) |
|  |  |  |  | χ^2^=76378.58, *P*=0.000; I^2^=99.7% |
| Upper-middle-income | 354 | 71,017 | 39,500,789 | 1.538 (1.441-1.636) |
|  |  |  |  | χ^2^=41318.94, *P*=0.000; I^2^=99.1% |
| Not stated | 3 | 19,625 | 11,257,164 | 1.841 (0.988-2.693) |
|  |  |  |  | χ^2^=1784.45, *P*=0.000; I^2^=99.9% |
| **Monitoring models (*χ*^2^=14.03, *P*=0.001）** | | |  |  |
| Hospital-based model | 565 | 192,239 | 73,943,900 | 2.441 (2.332-2.549) |
|  |  |  |  | χ^2^=120750.86, *P*=0.000; I^2^=99.5% |
| Population-based model | 52 | 9,695 | 3,017,454 | 3.373 (2.897-3.848) |
|  |  |  |  | χ^2^=6664.51, *P*=0.000; I^2^=99.2% |
| **Gender (*χ*^2^=40.955, *P*=0.000）** | | |  |  |
| Male | 30 | 14,910 | 4,333,084 | 4.175 (3.524-4.825) |
|  |  |  |  | χ^2^=5637.55, *P*=0.000; I^2^=99.5% |
| Female | 30 | 11,957 | 3,758,334 | 3.533 (2.927-4.140) |
|  |  |  |  | χ^2^=4823.63, *P*=0.000; I^2^=99.4% |
| **Urban and rural areas (*χ*^2^=1668.676, *P*=0.000）** | | | |  |
| Urban | 25 | 13,375 | 3,240,290 | 3.416 (2.547-4.285) |
|  |  |  |  | χ^2^=6177.63, *P*=0.000; I^2^=99.6% |
| Rural | 25 | 8,346 | 3,545,086 | 2.582 (2.053-3.111) |
|  |  |  |  | χ^2^=4516.41, *P*=0.000; I^2^=99.5% |

CHD: congenital heart disease; CI: confidence interval; TSD: test for subgroup difference.
