# Supplementary material for: Birth prevalence of congenital heart disease in China, 1980–2019: a systematic review and meta-analysis of 617 studies
Source: Eur J Epidemiol. 2020 Jun 9;35(7):631–42. doi: 10.1007/s10654-020-00653-0 (PMC7387380; doi:10.1007/s10654-020-00653-0)
Supplement: Supplementary file 13 — Supporting information file 1 (DOCX 13 kb) [file 10654_2020_653_MOESM13_ESM.docx]

Supporting Information Table 1: 21 included CHD subtypes and the classification [based on Hoffman's method ^[11]^ (2002) and modified according to methods from Zhao QM ^[12]^ (2013)].

| Severe CHD | Subtypes | Abbreviations | ICD 10 Code |
| --- | --- | --- | --- |
| Cyanotic lesions | 1. Tetralogy of Fallot | TOF | Q21.3 |
|  | 2. D-transposition of the great arteries | TGA | Q20.3 |
|  | 3. Double outlet right ventricle | DORV | Q20.1 |
|  | 4. Single ventricle | SV | Q20.4 |
|  | 5. Interrupted aortic arch | IAA | Q25.2 |
|  | 6. Truncus arteriosus | TA | Q20.0 |
|  | 7. Hypoplastic left heart syndrome | HLHS | Q23.4 |
|  | 8. Total anomalous pulmonary venous return | TAPVR | Q26.2 |
|  | 9. Right heart lesions |  |  |
|  | a. Tricuspid atresia | TA/TS | Q22.4 |
|  | b. Pulmonary atresia | PA | Q22.0 |
|  | c. Ebstein anomaly | EA | Q22.5 |
| Acyanotic lesions | 1. Atrioventricular septal defect | AVSD | Q21.2 |
|  | 2. Endocardial cushion defect | ECD | Q21.2 |
|  | 3. Mitral insufficiency/ regurgitation | MR | Q23.3 |
|  | 4. Dextrocardia | Dextrocardia | Q24.0 |
| Mild CHD | Subtypes | ICD 10 Code | ICD 10 Code |
| Cyanotic lesions | Pulmonary stenosis | PS | Q25.6 |
| Acyanotic lesions | 1. Ventricular septal defect | VSD | Q21.0 |
|  | 2. Atrial septal defect | ASD | Q21.1 |
|  | 3. Patent ductus arteriosus | PDA | Q25.0 |
|  | 4. Coarctation of the aorta | CoA | Q25.1 |
|  | 5. Aortic stenosis | AS | Q25.3 |
